# Characterization of minimal lesions related to the presence of visna/maedi virus in the mammary gland and milk of dairy sheep

**DOI:** 10.1186/s12917-019-1855-3

**Published:** 2019-04-10

**Authors:** E. Gayo, L. Polledo, A. Magalde, A. Balseiro, M. J. García Iglesias, C. Pérez Martínez, S. Preziuso, G. Rossi, J. F. García Marín

**Affiliations:** 1 Veterinary Pathology Unit, School of Veterinary Medicine, University of León, León, Spain; 2 Micros Veterinaria, León, Spain; 3 SERIDA, Gijón, Spain; 4 School of Bioscences and Veterinary Medicine, University of Camerino, Camerino, Italy

**Keywords:** Histopathology, Lentivirus, Mammary gland, Milk, Minimal lesions, Sheep, Visna maedi

## Abstract

**Background:**

In order to characterize the complete range of lesions, especially minimal, affecting mammary gland and viral antigen distribution and target cells using immunohistochemistry in naturally Visna/maedi (VM) 84 infected sheep were studied, forty-four from flocks with clinical cases (A) and 35 randomly sampled from two abattoirs (B) together with five negative controls (C). An immunocytochemistry technique was developed and further milk samples (*n* = 39) were used to study viral excretion, carrier cells and the role of milk and colostrum in the transmission of the disease.

**Results:**

All sheep from group C and three sheep from group B were negative to VM in tissue sections by histopathology, immunohistochemistry and PCR, and also in serum using ELISA. Several degrees of CD3 + lymphocytic interstitial mastitis were observed in groups A and B: minimal (+) *n* = 26 sheep; moderate (++), *n* = 32 and severe (+++), *n* = 12. No differences in lesion distribution were observed between groups A and B. Viral presence was confirmed by immunohistochemistry using two different antibodies and/or PCR in every tissue with lesions while serology was negative in six sheep with lesions. Two milk samples taken from milk tanks from two flocks from group A and fourteen milk samples from 29 infected sheep from group B were positive to VM (most of them from animals with moderate and severe lesions). Positivity was only found in macrophages, even in focal and minimal lesions, while no positivity was observed in epithelial or any other cells in either tissue and milk samples.

**Conclusions:**

This new observation of the minimal lesions described in this work increased the prevalence of VM lesions in mammary gland up to 90.9% and VM should be considered as a differential diagnosis when minimal interstitial lesions are detected. A high prevalence of VM was observed in intensive milk-producing sheep, ELISA serology did not detect as positivity all infected animals, while histology, IHC or PCR showed higher sensitivity. The cytological technique developed was very useful in milk-cell studies using hematoxylin and eosin and immunocytochemistry. Viral detection in milk samples (16/39) confirms a potential but limited role of milk/colostrum in viral transmission.

**Electronic supplementary material:**

The online version of this article (10.1186/s12917-019-1855-3) contains supplementary material, which is available to authorized users.

## Background

Ovine Visna Maedi (VM) is caused by Visna/maedi virus (VMV), which together with caprine arthritis-encephalitis virus are small ruminant lentiviruses (SRLV) [1]. Clinically, it is a slow progressing disease characterized by a gradual loss of body condition due to chronic inflammation in different organs [2–4] consisting of interstitial pneumonia, interstitial mastitis, non-purulent meningoencephalitis with demyelination of the CNS also affecting spinal cord and rarely arthritis [4–6]. The mammary syndrome is difficult to detect and mainly consists of mammary gland induration, milk production loss and deficient lamb growth [7]. Flock seroprevalence in Spanish Assaf dairy sheep kept under indoor intensive farming system is estimated at 77%, up to 98% in northern Spain [8, 9], which is the most frequent management system in this study area and causes important economic loses [10]. To date, adequate monitoring programs have proved to be the only tool to control the infection as no treatments or vaccines are available [11].

Monocytes/macrophages and dendritic cells are the main target cells of SRLV. These target cells could migrate to regional lymph nodes from where viral systemic dissemination could occur [12–14] or could reach the bone marrow infecting myeloid precursor cells and reaching the bloodstream [15, 16], carrying the viral DNA into blood with minimum transcription until the maturation of monocytes into macrophages in the tissue.

The presence of virus in macrophages from mammary gland and milk or colostrum from infected sheep has been reported [17–20] and thus the possibility of infection of new-born lambs by ingestion of infected colostrum [20]. This fact has also been considered in CAEV infected animals, where colostrum has been proposed as one of the most important transmission routes [21, 22]. However, the importance of milk and colostrum in the spread of the disease in sheep under natural conditions is a continuous source of debate. The lactogenic route of transmission has been described as important in enzootic infections [16, 23], while other studies suggest that its contribution to the spread of the disease seems to be low compared to horizontal/respiratory transmission [24–26].

SRLV presence in the nuclei of cells resembling mammary glandular epithelial, endothelial and fibroblast-like cells using immunohistochemistry (IHC) [27] or using in situ hybridization [27, 28] has been described.

The aims of this study are (1) to examine the cell types infected and the distribution of VMV antigen in healthy and injured areas of infected mammary gland, especially in minimal lesions and its relationship with blood vessels in order to better understand the pathogenesis of the disease in this target organ, both in animals from flocks with clinical signs or randomly selected from abattoirs and the possible role in the transmission of the disease; (2) to develop a simple and suitable immunocytochemistry (ICC) technique to identify VMV antigen in different somatic milk cells and (3) to study viral excretion using PCR in milk and ICC in milk cells with the aim of clarifying the role of milk in the transmission of the infection and its possible application in the diagnosis and control of this disease. (4) The final aim of this work is to evaluate the diagnosis techniques and virus identification in tissues and milk, which could be of vital importance.

## Results

**Fig. 1:**
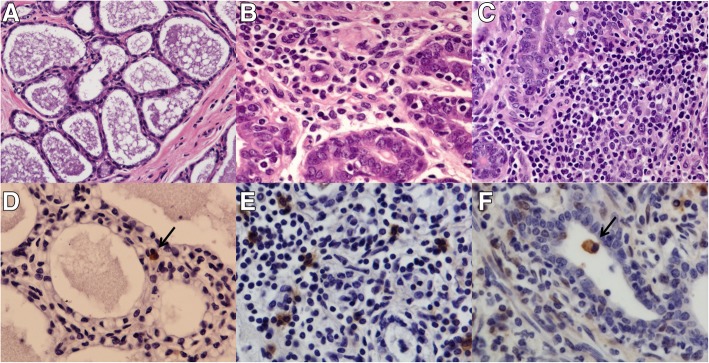
Visna maedi (VM) lesions in udder sections. **a**: healthy mammary gland. No inflammatory infiltrate is observed. HE. 20X **b**: moderate lesions (++). Presence of inflammatory cells in mammary interstitium and within epithelial cells.40X **c**: severe lesions (+++) with high quantity of lymphocytes and macrophages producing acini destruction. 40X **d**: anti-gp135 immunohistochemistry in udder with minimal lesions. Black arrow indicates a positive macrophage-like cell within the intertitium with a few inflammatory cells in the surroundings. 40X. **e** anti-p28 of CAEV/VMV immunohistochemistry in udder samples with severe lesion (+++). 40X. **f**: positive cell in the acinar lumen. No positivity is observed in epithelial cells

Three of 35 sheep from group B (8.6%) and all five animals from group C showed no lesions and were negative in IHC and PCR in tissue and milk samples and ELISA in sera and thus were considered as being not infected (Fig. 1A). Viral infection was confirmed using IHC (Figs. 1B-D), PCR and/or ELISA in the remaining animals which were considered VM infected.

### Histopathology

Interstitial mastitis was observed in 40 samples of group A (90.91%) [29] and in 30 samples from group B (85.7%). Minimal lesions (+) were observed in 26 infected animals (26/76, 34.2%), moderate lesions (++) in 32 infected sheep (32/76, 42.1%) and severe lesions (+++) in 12 infected sheep (12/76, 15.8%) with no large differences in lesion severity distribution between groups A and B (Table 1). Inflammatory infiltrates were always located in the interstitium between acini in every type of lesion. Differences in the severity of the inflammatory infiltrate and lymphoid hyperplasia were observed in different mammary lobules. These differences were even observed between adjacent acinis, especially in minimal lesions, with most of them being healthy. No lesions were observed in six infected sheep (6/76, 7.9%) which showed no inflammatory cells in the interstitium and only a few and focal small lymphocyte aggregates were found in association with canaliculi.

**Table 1:** Occurrence of lesions due to VM in terms of intensity of lesions and comparison between groups A and B

Grade of lesions	Group A	(%)	Group B	(%)	Total	(%)
Minimal (+)	15	(34.1)	11	(31.4)	26	(32.9)
Moderate (++)	18	(40.9)	14	(40)	32	(40.5)
Severe (+++)	7	(15.9)	5	(14.3)	12	(15.2)
None (−)	4	(9.1)	5	(14.3)	9	(11.4)
Total	44		35		79	

### Immunohistochemistry

The predominant cell type within the interstitial inflammatory infiltrate was CD3+ with some scattered CD79+ and CD68+ cells, regardless of the severity and extension of the lesions. Macrophages were observed in different quantities independent of the predominance of CD3+ and were found not only in the interstitium but also between epithelial cells or in the acinar lumen.

All sheep from group A and 32 sheep from group B were positive to SRLV using IHC with both antibodies while the eight sheep considered not infected were negative (Table 2). Positivity against SRLV was only found in macrophages in mammary tissue, even in focal and minimal lesions, while no positivity was observed in healthy mammary tissues in the same animals and in sheep with no lesions. SRLV antigen was found around blood vessels in minimal lesions while in moderate and severe lesions it was present in perivascular cuffs, within the epithelium and even in cells located in the acinar lumen in eight sheep (Fig. 1). Positive cells were also observed isolated within the interstitium, associated with minimal or moderate lesions showing no IHC sign in close areas and in intralobular and interlobular connective tissue. No positive signal was present in epithelial cells or other cell types using either monoclonal anti p28 and polyclonal anti gp135 antibodies (with the exception of very sporadic and doubtful positivity fibroblast-like cells in the interstice). No immunolabelled cells were detected in sections used as negative controls but labelling was invariably seen in positive control sections.

**Table 2:** histopathology (HE), IHC and PCR results udder (u) and milk (m) samples and ELISA results in infected and non-infected sheep. *2/10 samples were from milk tank and 7/26 were from individual sheep

Group	HE (%)	IHC (u) (%)	PCR (u) (%)	ICC(m) (%)	PCR(m) (%)	ELISA (%)
Infected	70/76 (92.1)	76/76 (100)	72/76 (94.7)	*9/36 (25)	13/26 (50)	53/62 (85.5)
Not infected	0/8 (0)	0/8 (0)	0/8 (0)	0/8 (0)	0/8 (0)	0/8 (0)

### Polymerase chain reaction in tissue samples

Forty animals from group A and 32 from group B were positive using PCR to detect VMV (Table 2). Three of the four negative animals from group A showed minimal lesions and the remaining sheep showed moderate lesions. The three negative animals from group B were those considered as being not infected. No positivity was observed in PCR against *Mycoplasma spp*.

### Serology

Thirty-nine animal from group A were seropositive (88.6%), three had inconclusive results (OD of 0.4 ± 0.1), and two were negative. [29] Fourteen infected sheep from group B were seropositive (77.8%) while four were negative: two showed minimal lesions and one moderate lesions (Additional file 1: Tabe S1).

### Cytological studies in milk

**Fig. 2:**
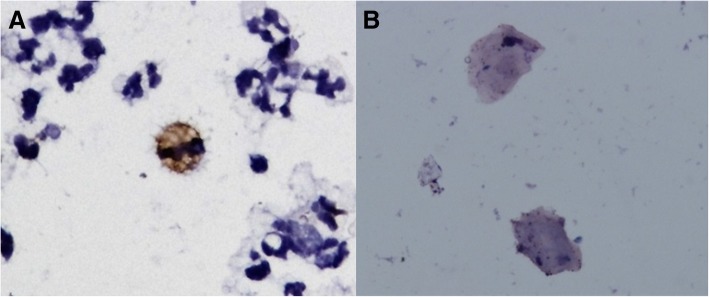
Anti-p28 of VMV/CAEV immunocytochemistry in milk samples. 60X. **a**: positive macrophage-like cell surrounded by some neutrophils and lymphocytes with no IHC sign. **b**: negative epithelial cells

HE and May-Grunwald Giemsa staining allowed for a specific identification of the different milk cell populations (Fig. 2a). Macrophages and neutrophils were easily identified using HE staining, while the May-Grunwald Giemsa method was better for the identification of epithelial cells.

After milk cytocentrifugation cells appeared distributed on the slide in three concentric cellular rings: neutrophils were the predominant cell population in the centre, epithelial cells were the most abundant cells in the periphery, and macrophages were distributed homogeneously.

### Immunocytochemistry in milk samples

Positive macrophage cells were observed using ICC in milk from the two cistern milk samples and in seven individual milk samples (26.9%) (Table 2) (Fig. 2b). Three of them were from sheep with severe lesions and four from sheep with moderate lesions (Additional file 1: Table S1). This labelling appeared as a brownish deposit in the cytoplasm of macrophages and no positivity was observed in epithelial-like cells or others cells. ICC slides showed no fat globules, unlike the cytology slides stained with HE or May-Grünwald-Giemsa, which could be attributed to the successive washings in PBS and the blocking of the endogenous peroxidase activity with hydrogen peroxide 3% in distilled water for 30 min.

### Polymerase chain reaction in milk samples

Milk samples from 13 sheep were positive to VMV by PCR (50%). Four of them showed severe lesions, seven showed moderate lesions and two minimal lesions. Six of them were also positive using ICC. (Table 2) (Additional file 1: Table S1).

#### Diagnosis techniques comparison

IHC was the most sensitive technique with 100% positivity in groups A and B. Histopathology detected 92.1% infected animals, with 90.9% detection in group A and 93.8% in group B. ELISA showed 88.6 and 77.8% positive values in groups A and B, respectively (85.5% of infected animals). PCR was more sensitive than ELISA in VM viral detection with values increasing to 90.9% in group A and 100% in group B (94.7% infected animals). PCR was also more sensitive than ICC in milk samples, with values increasing from 25 to 50% for viral detection, respectively (Table 2).

## Discussion

Detailed characterization of udder lesions and VMV antigen distribution and target cells, especially in minimal lesions, contributed to a better understanding of the pathogenesis of VM infection in this target organ in naturally infected milk-producing sheep. VM has been reported as the major cause of sheep culling in two intensively-managed dairy sheep flocks in the same study area and has an important economic impact [10]. Here, the percentages of lesions of different severity were similar in sheep from known infected flocks and randomly selected from two different abattoirs. Fifty-seven per cent of animals from infected flocks and 54% of sheep from the abattoir showed moderate or severe lesions which confirm the importance of the mammary form of the disease in dairy sheep culling and its relationship with production loss. Serological results also highlight the high prevalence of VM infection in intensive milk-producing sheep in Castilla y León (Spain), with 91.4% PCR and IHC-positive sheep from abattoirs, similar to previously reported seroprevalence [9]. Serology did not detect any infection in six sheep with positive histopathology, IHC and PCR results while diagnosis of all seropositive sheep was confirmed using histopathology, IHC and/or PCR. IHC and PCR clearly increased VM prevalence in infected sheep reaching 100 and 94.7% positivity respectively compared with 85.5% seropositivity with the ELISA test. The lower sensitivity obtained with PCR compared with IHC could be related to DNA degradation after formalin fixation of the samples from animals of group A or to low viral presence [32]. However, a PCR sensitivity higher than 90% in these animals point towards a high level of conservation of proviral VMV DNA in formalin-fixed and paraffin-embedded samples, which are the only available material in many retrospective studies. These results agree with previous studies where VMV positivity was described using PCR in formalin fixed and paraffin-embedded samples even after 30 days of fixation [33].

The higher sensitivity of PCR (50%) compared with ICC (25%) in milk samples could be due to the difficulty of macrophage detection in the cytological smear, especially in those positive to VMV. The existence of one milk sample positive to VMV in ICC but negative in PCR could be related to a low number of infected macrophages or a low viral load in this particular sample, which has been described as one of the main concerns of VMV detection using PCR [32]. Viral detection with this technique even in animals with minimal lesions would confirm it usefulness for VMV detection.

More than one diagnosis technique should be performed in order to detect all infected animals. IHC in tissues would be used as a gold standard technique for disease detection, being the only method capable of detecting all VM infected sheep. Using only the ELISA test for VM diagnosis, as frequently happens under field conditions, involves the risk of obtaining false negative animal related with lymphocytic type lesion in the immunopathological spectrum of VM [30]. Moreover, in this work the false negative animals are also associated to minimal lesion probably with no relevant response detectable in serum samples. As has also been observed in this work, this feature is very important in flocks submitted to control programs where VM prevalence is always lower and detection of every single infected sheep, even those with minimal lesions, is of great interest for the eradication of the disease.

Two infected sheep with minimal lesions and one with no lesions showed negative ELISA values and positive IHC and PCR results. This finding agrees with the hypothesis of minimal lesions would be related to initial or latent stages of the disease where the number of inflammatory cells would not be enough to develop a local immune response and antibody production detectable in serum samples using current techniques. [30]. Thus, after VMV infection viral presence in tissue detectable using PCR and IHC would be before serological response, as probably due to the fact that the development of lesion and local immune response are poor and thus not detectable in serological test. Negative ELISA test results in animals with moderate or severe lesions may be related to the development of a lymphocytic pattern [29, 34] more frequent in the mammary gland, as has been recently published [29].

Detection of minimal lesions clearly increased the prevalence of VM lesions in mammary gland confirming previously published results [29]. Minimal inflammatory infiltrates and subepithelial lymphoid aggregates have been described in healthy udders on the limit between the teat duct and cistern and have been related to the protection in the early stages of bacterial invasion [35]. Those minimal infiltrates were not found in non-infected animals or in non-affected tissue of infected sheep in this study. This lack of inflammatory infiltration could be explained by the selection and the constant microbiological and somatic cells controls performed in intensive milk-producing flocks which is the management system used for all dairy flocks of Spanish Assaf breed in the region studied. The negative result for the presence on *Mycoplama spp.* could confirm this hypothesis.

The predominance of a CD3+ lymphocytic response in animals from group B agrees with our previous description in group A [29]. However, it contrasts with findings in CNS and lung where two lesion pattern (histiocytic and lymphocytic) were described regarding the predominant inflammatory cell population [29, 34], which could confirm a different local immunological response in this target organ. Nevertheless, the presence of more macrophages within the inflammatory infiltrate in the mammary gland of some animals may be related to the development of a histiocytic or lymphocytic pattern with the previously mentioned differences.

The presence of IHC-positive signal for p28 and gp135 of SRLV in macrophage in mammary gland and milk agrees with previous results in different VM target organs [7]. Presence of surface *env*-encoded protein gp135 together with nucleocapsid *gag*-encoded protein p28 and PCR positive in the same sample, indicates not only viral presence but also active infection with the presence of different virus stages in the same cells. In most cases viral presence was associated with inflammatory cells and p28 and gp135 proteins of SRLV were not observed in non-affected areas of infected tissues. This fact, together with the viral distribution around blood vessels in minimal lesions and spread to interstitial mammary tissue in moderate and severe lesions, agrees with the hypothesis of viral invasion via monocytes/macrophages [29, 36, 37]. Infection would begin in very focally concrete locations of the organ, related with different blood vessel, and then would spread to the rest of the tissue. IHC positivity to VMV in isolated cells located in areas with no lesions or in connective tissue in some samples could be related to small blood vessels not clearly observed in these particular histological sections.

SRLV immunoreactivity was not observed in epithelial cells using both antibodies against gp135 and p28 proteins in contrast to previous descriptions [27] and to the hypothesis of the possible SRLV replication in epithelial cells “in vivo” as has been described “in vitro” [27, 38]. Furthermore, in situ hybridization or PCR as previously reported in epithelial cells of mammary gland [27, 28] showed the presence of viral nucleic acids but not the presence of viral proteins or active viral replication. Nevertheless, this study, by using two different antibodies against surface and nucleocapsid proteins of VM, shows that active infection occurs as previously mentioned. However, further studies into viral replication in mammary gland should be conducted.

The presence of SRLV antigen in all mammary glands with lesions including cells in acinar lumen in some cases and in milk cells confirm the potential transmission of the disease by lactation. However, the small number of positive cells and few positive milk samples by IHC and PCR could highlight the limited potential of transmission as it has also been proposed by others authors, [39] leading to a higher VM transmission risk by continuous lactation and not only by colostrum ingestion. Most positive milk samples came from sheep with moderate or severe lesions in udder (85.7%) and a higher number of positive cells in tissue by IHC as well as positive PCR results. These findings support that reaching canaliculi is easier for infected macrophages when lesions are more severe and widespread than when they are focal and limited to perivascular cuffs.

The ICC technique developed in milk in this study allows for the obtaining of well-preserved monolayer cell smears and optimal identification of cell populations and VMV antigen in somatic milk cells. After the first centrifugation, cell distribution within the Falcon tube are very likely conditioned by their density as happens in blood vessels during inflammation [40]. The optimum drying time of the cytology after cytocentrifugation was 20 min, much shorter than the three hours used in previous studies [41]. HE staining is very useful in identifying leukocytes while epithelial cells were better identified with May-Grünwald-Giemsa staining [42]. This latter staining helps to differentiate these cells from artefacts, giving a different pink colour compared with artefacts or other cells populations. The epithelial cells show normal morphology without abnormalities, suggesting lack of mammary lesions absence of hyperplasia or neoplasia in the mammary gland epithelia of the sheep studied [42].

## Conclusions

Detection of minimal lesions using histopathology increased the incidence of VMV in mammary gland. Viral antigen distribution from a few cells in perivascular cuffs to the rest of tissue in moderate and severe lesions confirm viral progression from blood monocytes to macrophages in this target organ. SRLV immunoreactivity was only observed in macrophages and not in epithelial cells. The IHC in tissues can be used as a gold standard technique for disease detection, being the only one that detected all VM infected sheep. Virus detection in milk samples using ICC and PCR confirms the potential but limited role of milk/colostrum in viral transmission and point towards other more important transmission routes.

## Methods

### Animals

A total of 84 udder samples from adult Spanish Assaf sheep from a region of Spain (Castilla y León) were studied (Additional file 1: Table S1).Forty-four sheep (Group A) were previously described. They were selected from those submitted for routine necropsy examination and histopathology diagnosis to the Pathology Diagnostic Service of the Veterinary School (León, Spain). These animals had died naturally on the farm or were culled due to evident deterioration of their health status and humanly euthanized coming from seropositive flocks with clinical cases and diagnosis of the disease. These animals were 1–3 years of age and came from six different SRLV infected intensive milk-producing flocks of 300–1200 sheep subjected to SRLV control and with highly qualified staff [29, 30]. Additionally, 35 adult sheep (Group B) with unknown clinical histories were randomly sampled from two abattoirs during regular slaughtering. Further samples of five sheep (Group C) from a free-SRLV flock were used as negative controls. None of sheep selected for this study showed gross or histological mastitis findings of bacterial or fungal origin.

### Tissue samplings, histopathology

Tissue samples from all 84 sheep were systematically taken from glandular parenchyma and udder cisterns. Samples were fixed in 10% neutral buffered formalin for 48 h at room temperature and were then embedded in paraffin wax. Serial sections (4 μm) were made and stained with hematoxylin and eosin (HE) and examined using light microscopy. Lesions were classified, without having previous knowledge of other results such as minimal (+), moderate (++) and severe (+++) as previously described [29, 30].

### Milk samplings

Cistern milk samples from two flocks of group A (*n* = 10) with 1267 × 10^3^ and 591 × 10^3^ somatic cells/ml and 60 and 63% VM seroprevalence respectively and individual milk samples from group B (*n* = 29) and C (*n* = 5) were taken to better cytological, immunocitochemical (ICC) and PCR studies.

### Cytology optimization in milk samples

In order to optimize the procedure, the following variables on the protocol were considered: centrifugation speed, centrifugation time, volume of the samples, drying and fixation times. Tank milk samples (n = 10) of 12 ml were taken in Falcon tubes from two seropositive flocks of group A. After milk centrifugation (Orto® and p-Selecta®) the largest number of cells was identified at 1500 rpm. Approximately three 500 μl samples were taken in 1 ml vial from three different locations (top, middle and bottom of the Falcon tube) in order to locate the region with higher cell concentration and less fat globules. These three samples were subdivided in different volumes and cytocentrifuged (Cytospin 3®) using different centrifugation times at 1000 rpm following the manufacturer’s instructions. Smears obtained from the top of the Falcon tube and cytocentrifugation at 1000 rpm for four minutes was established as the best combination. Different drying times after cytocentrifugation and fixation times with methanol at room temperature were tested and were established at 20 min each.

This technique was carried out in milk samples from the 29 sheep from group B and in further ten tank milk samples from the two infected flocks of group A after optimization. Samples were stained with hematoxylin and eosin (HE) and with May-Grunwald-Giemsa and were examined using light microscopy.

### Immunohistochemistry and immunocytochemistry

Immunohistochemistry in tissue samples: Further serial sections (4 μm) were prepared for IHC. The following antibodies were used as previously described [29]: polyclonal anti-CD3 for T cells (Dako, Denmark); monoclonal anti-CD79 for B cells (Dako, Denmark); monoclonal antibodies anti-CD68 for macrophages (Dako, Denmark), and monoclonal anti-p28 of VMV/CAEV (VMRD, USA) for viral detection. Further polyclonal anti-gp135 of SRLV elaborated by our research group and diluted 1:30000 was used in all samples. In order to obtain rabbit anti-serum against the VMV, a New Zealand white rabbit was immunized with gp135 protein supplied by Pourquier (1.6 g) emulsified with 1 ml of complete Freund’s adjuvant on days 1, 14, 21 and 28. The last boost containing the gp135 protein in the absence of Freund’s adjuvant was administered intravenously on day 35. The serum on day 38 and the pre-immune serum from the same rabbit were used for immunohistochemical studies. Sensitivity and specificity of the technique were tested in ten known VM positive and negative samples, respectively.

Immunocytochemistry performed in milk samples: The optimized smear preparation was used for ICC in milk samples from the 29 sheep from group B and in ten tank milk samples from the two infected flocks of group A. Anti-p28 and anti-gp135 for SRLV antibodies described above were used [29].

### Polymerase chain reaction in tissue and milk samples

Tissue samples from all sheep from group B were frozen for PCR studies. DNA was extracted using Genomic DNA Isolation Kit and the DNA eluted in a final volume 100 μl (NORGEN, Biotek Corporation). A nested PCR protocol for amplifying a sequence of the LTR gene of VMV [12] and a PCR protocol to exclude *Mycoplasma spp* infection [31] were used in both groups A (DNA previously extracted from paraffin embedded tissue samples [29]) and B. Mammary gland DNA samples from sheep infected with VMV Pi130 and DNA from *Mycoplasma agassizii* were used as positive controls in PCR*,* while water instead of DNA was used as negative control in each PCR run.

White milk cells were extracted (centrifugation of 15 ml for 10 min at 2000 rpm at 10 °C and fat removed with micropipette × 3; centrifugation of 1.5 ml for 10 min at 1400 rpm at 10 °C and washed with PBS × 3) and pellets were frozen for PCR studies.

### Serology

ELISA (ELITEST®) for detection of SRLV antibodies was performed in animals from group A (*n* = 44), group B (*n* = 19) and group C (*n* = 5) as previously described [30].

## Additional file

Additional file 1: **Table S1.** histology, ELISA, IHC and PCR results in udder (U) and milk (M) samples. NS: no sample available. Animals highlighted in red were considered as not infected. (XLSX 13 kb)

## Abbreviations

CAEV: caprine arthritis encephalitis virus
CNS: central nervous system
HE: hematoxylin eosin
ICC: immunocytochemistry
IHC: immunohistochemistry
PCR: polymerase chain reaction
SRLV: small ruminant lentiviruses
VM: visna maedi
VMV: visna/maedi virus
